# Audit of compliance with WHO surgical safety checklist (modified for electroconvulsive therapy including NPSA advice)

**DOI:** 10.1192/bjo.2021.213

**Published:** 2021-06-18

**Authors:** Faisal Alam, Rizwan Ashraf, Kyaw Sein, Terri Feeney

**Affiliations:** GMMH NHS Foundation Trust

## Abstract

**Aims:**

This audit aims to evaluate the compliance with the WHO surgical safety checklist during the electroconvulsive therapy treatment in ECT clinic at Greater Manchester Mental Health Bolton Directorate. The audit is based on WHO surgical safety checklist modified for ECT including National Patient Safety Agency advice. The goal is to improve the compliance and in turn improve clinical outcomes.

**Background:**

The WHO surgical safety checklist (modified for Electroconvulsive therapy including NPSA advice) is devised to promote patient safety, improve teamwork, reduce errors/adverse events and improve overall quality of care. An audit was completed regarding the compliance with the safety checklist at the Bolton ECT clinic and to assess how this could be improved.

**Method:**

Following approval from the clinical audit department, GMMH NHS Foundation Trust, 20 checklists from randomly selected patient ECT files were included in this audit. We looked at whether the checklists were completed, signed and dated. Our current WHO surgical safety checklist is as per the Electroconvulsive therapy accreditation service standards.

**Result:**

A total of 20 WHO surgical safety checklists were reviewed. 95% of the checklists (19/20) were completed by the duty Psychiatrist. 1 form was not completed. 25% (5/20) were not signed rendering them invalid. A total of 75% checklists were complete and valid. Checklists were present in all the case notes.

**Conclusion:**

Compliance with the WHO surgical safety checklist during the electroconvulsive therapy treatment can be challenging due to various reasons ranging from time pressure to difficult clinical situation. This audit has highlighted that the overall compliance with the set standards (100% completion) was not achieved. A repeat audit will be important to further improve the compliance and overall clinical outcome.

